# Effects of treatment with janus kinase inhibitors on coronary microvascular perfusion in patients with rheumatoid arthritis: an observational prospective cohort study

**DOI:** 10.1007/s00296-025-05862-y

**Published:** 2025-04-19

**Authors:** Panagiota Anyfanti, Elena Angeloudi, Eleni Pagkopoulou, Maria Boutel, Georgia-Savina Moysidou, Kleopatra Deuteraiou, Eleni Bekiari, Michael Doumas, George D. Kitas, Theodoros Dimitroulas

**Affiliations:** 1 Third Department of Internal Medicine, Papageorgiou Hospital, Aristotle University of Thessaloniki, Thessaloniki, Greece; 2 Second Medical Department, Hippokration Hospital, Aristotle University of Thessaloniki, Thessaloniki, Greece; 3 Fourth Department of Internal Medicine, Hippokration Hospital, Aristotle University of Thessaloniki, Thessaloniki, Greece; 4 Rheumatology-Clinical Immunology Unit, 4th Department of Internal Medicine, Attikon University Hospital, National and Kapodistrian University of Athens Medical School, Athens, Greece; 5 2nd Propedeutic Department of Internal Medicine, Hippokration Hospital, Aristotle University of Thessaloniki, Thessaloniki, Greece; 6 Department of Rheumatology, Russells Hall Hospital, Dudley Group NHS Foundation Trust, Dudley, UK; 7 School of Sport, Exercise and Rehabilitation Sciences, University of Birmingham, Birmingham, UK

**Keywords:** Rheumatoid arthritis, Janus kinase inhibitors, Myocardial perfusion

## Abstract

Despite their increasing use and their proven efficacy in the treatment of rheumatoid arthritis (RA), Janus kinase (JAK) inhibitors have been questioned by credible cardiovascular safety concerns. To date, mechanistic links of cardiovascular complications to JAK inhibitors remain largely unexplored. We aimed to investigate the effect of JAK inhibition on coronary microvascular blood flow in a previously published cohort of treated patients with RA. We prospectively enrolled RA patients initiating treatment with JAK inhibitors. Study procedures were performed at baseline and repeated 3 months after treatment. Patients underwent applanation tonometry in the radial artery to assess subendocardial viability ratio (SEVR) otherwise known as Buckberg index, a noninvasive marker of myocardial perfusion. Thirteen patients with RA were enrolled, of whom 11 completed the study. All patients presented with at least one cardiovascular risk factor (e.g., age ≥ 65 years, history of current or past smoking, obesity, hypertension). No change in other than antirheumatic treatment was performed during the study, and no significant changes were observed in baseline characteristics other than triglyceride levels. Compared to baseline, three months treatment with JAK inhibitors did not significantly alter SEVR values [126 (102-144) % vs. 134 (106-251) %, *p* = 0.083]. Three months treatment with JAK inhibitors did not seem to significantly affect myocardial perfusion in a small RA cohort with cardiovascular risk factors, who would be presumably more vulnerable to adverse treatment-related cardiovascular effects. Larger studies with longer follow-up are needed to document the effects of JAK inhibitors on the myocardium.

## Introduction

Janus kinase (JAK) inhibitors are small-molecule drugs classified as biological disease-modifying antirheumatic drugs (DMARDs), that offer the unique advantage of an oral route of administration and have proven efficacy in the treatment of rheumatoid arthritis (RA) since the approval of tofacitinib for these patients in 2012. However, the ORAL Surveillance study demonstrated that risks of major adverse cardiovascular events and cancers were higher in RA patients treated with tofacitinib and produced widespread concerns regarding their cardiovascular safety [1]. Post hoc analyses of the trial data revealed that factors associated with increased risk of these adverse events included age ≥ 65 years, high cardiovascular risk at baseline, smoking, active RA and suboptimal treatment of cardiovascular comorbidities [2]. The 2022 updated European League Against Rheumatism and America College of Rheumatology (EULAR) recommendations for the management of RA with synthetic and biological DMARDs incorporated these concerns by stating that risk factors must be considered when intending to prescribe a JAK-inhibitor, including cardiovascular (age ≥ 65 years, history of current or past smoking, other cardiovascular risk factors such as obesity and hypertension) and thromboembolic risk factors [3]. Almost simultaneously, European Medicines Agency (EMA) issued safety warnings in November 2022, suggesting that JAK-inhibitors should be used in high-risk patients only in the absence of no suitable treatment alternatives [4]. Additionally, Food and Drug Administration (FDA), called for alert about increased risk of serious heart-related events, cancer, thrombotic episodes thus death incidents for patients that will start or are already in treatment with several JAK-inhibitors and advised health providers to weight risks and benefits of such therapy [5].

Understanding the mechanistic links of potential adverse cardiovascular outcomes related to the use of JAK inhibitors could facilitate the identification of most suitable candidates for treatment with JAK inhibitors, i.e., patients with the minimal risk and the maximum benefit from treatment with these agents. Although the above safety issues might have had an impact on prescription rates of JAK inhibitors [6], the exact mechanisms of postulated adverse cardiovascular effects remain largely unspecified. Direct effects of JAK inhibitors on coronary microcirculation might provide a mechanistic link to the increased cardiovascular risk reported in previous observations, yet no study has evaluated this outcome to date.

Subendocardial viability ratio (SEVR) is a non-invasive marker of assessing endothelial dysfunction and coronary microvascular perfusion to subendocardium respectively. The potential clinical value of this index in association with surrogate markers of cardiovascular disease has been demonstrated in various populations, including patients with RA [7, 8, 9]. Analysis from two separate cohorts of RA patients revealed that SEVR was associated with markers of disease activity along with classical cardiovascular risk factors such as heart rate, high blood pressure and diabetes [10]. Further expanding this observation, lower SEVR was demonstrated in patients with RA in comparison with a control group, even in the absence of major cardiovascular risk factors and comorbidities [7, 8]. In patients with RA, SEVR was associated with galectin-3, a novel cardiac biomarker, and circulating dimethylarginines, biomarkers of endothelial dysfunction and atherosclerosis, although these associations faded after adjustment for inflammation and cardiovascular risk factors [8, 11]. Altogether, these results suggest that SEVR might have a role in cardiovascular risk prediction in RA.

To date, no study has investigated the impact of JAK inhibitors on coronary microvascular perfusion. We have previously shown that three-month treatment with JAK inhibitors did not affect macrovascular structure and function but induced significant microvascular alterations in the nailfold capillary network of treated patients [12]. We presently aimed to investigate whether JAK inhibition in the same cohort of RA individuals induced significant alterations in the coronary microvascular bed, by analyzing available data on patients’ myocardial perfusion, as assessed by SEVR.

## Methods

This was a prospective observational cohort study of a 3-month duration recruiting adult patients with RA who were eligible to receive JAK inhibitors (tofacitinib, baricitinib or upadacitinib) based on international recommendations (EULAR 2020) [13]. Patients who were unable to understand and sign the informed consent, had been previously treated with JAK inhibitors, presented with concomitant active malignancy or any disease with poor prognosis, recent cardiovascular event (myocardial infarction, unstable angina, stroke) within the past 6 months, and stage III-IV New York Heart Association (NYHA) heart failure. A detailed description of the study design and setting has been previously published [14]. All procedures were performed before administration of JAK inhibitors and were repeated 3 months later, whilst on treatment. The study was approved from the Institutional Review Board of Hippokration Hospital, Thessaloniki, Greece (protocol code 445/29-12-21, date of approval 17 February 2022). All participants gave written informed consent before inclusion in the study.

### Assessment of myocardial perfusion

Subendocardial viability ratio (SEVR), also known as Buckberg index, reflects the balance between myocardial oxygen supply and oxygen demand and is a valuable tool for evaluation of myocardial perfusion. Lower values of SEVR are indicative of poor perfusion of the subendocardium, which may lead to acute myocardial infarction in severe cases [9]. SEVR was calculated noninvasively with applanation tonometry in the radial artery (SphygmoCor device, AtCor Medical, Sydney, New South Wales, Australia). More specifically, the ratio of the area under the diastolic segment of the derived aortic pressure waveform (diastolic pressure time index, to the area under the systolic segment of the waveform (tension time index), which corresponds to the ratio of myocardial supply and demand, was calculated to derive SEVR values. Prior to the measurements, patients lied for 15 min in a quiet, temperature-controlled room and had abstained from caffeine, smoking, alcohol, and intense physical activity for at least 2 h.

### Statistical analysis

Statistical analysis was performed with the Statistical Package for Social Sciences, SPSS Inc., Chicago, IL, USA software, version 22. All data were analyzed after completion of the study. Descriptive statistical tests were used for categorical variables to present the cohort main characteristics, and data are expressed as median (minimum-maximum) values. Differences in outcomes at three months post-treatment to baseline were calculated with the non-parametric Wilcoxon signed-rank test. A probability value of *p* ≤ 0.05 was considered statistically significant.

## Results

The study enrolled 13 patients with RA who were started on upadacitinib (5 patients), baricitinib (4 patients) and tofacitinib (2 patients). Two patients dropped out before the end of the study and subsequently, 11 patients completed the study and were included in data analysis. Baseline characteristics of the study cohort have been previously published and are presented in Table 1 [12]. All patients presented with at least one cardiovascular risk factor. The majority (63.6%) were current smokers, 18.2% were ≥ 65 years old, 27.3% were hypertensives, 27.3% had dyslipidemia, and median body mass index was indicative of overweight. Patients suffered from long-standing disease, and median disease activity score in 28 joints (DAS28) was indicative of active disease.

**Table 1:** Baseline characteristics of the study population

	JAK inhibitors-treated patients (*n* = 11)
**Age (years)**	54 (47–65)
**Female: male ratio**	9:2
**Disease duration (years)**	13 (5–27)
**BMI (kg/m** ^**2**^ **)**	25.1 (19.7–32.3)
**Current smoking**,** n %**	7 (63.6)
**Hypertension**,** n (%)**	3 (27.3)
**Dyslipidemia**,** n (%)**	3 (27.3)
**DAS28**	3.9 (2.7–5.2)
**RF positivity**,** n (%)**	3 (27.3)
**Anti-CCP positivity**,** n (%)**	8 (72.7)
**Antirheumatic medication**	
Methotrexate, n (%)	4 (36.4)
Corticosteroids, n (%)	5 (45.5)
Biologics, n (%)	3 (27.3)
**Cardiovascular drugs**	
RAAS inhibitors, n (%)	3 (27.3)
Calcium channel blockers, n (%)	1 (9.1)
Beta blockers, n (%)	1 (9.1)
Diuretics, n (%)	2 (18.2)
Statins, n (%)	3 (27.3)

*JAK: janus kinase; BMI: body mass index; DAS28: disease activity score in 28 joints; RF: rheumatoid factor; anti-CCP: anti-cyclic citrullinated peptides; RAAS: renin–angiotensin–aldosterone system.*

*Continuous variables are presented as median (minimum-maximum) values.*

**Fig. 1:**
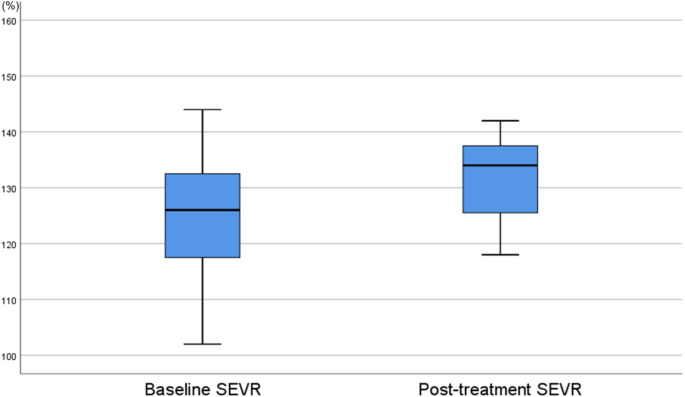
Change in SEVR from baseline to three-months treatment with JAK inhibitors in a prospectively studied sample of patients with RA

Participants’ body mass index, percentage of smoking, hypertension, and dyslipidemia, and concomitant medication remained unchanged throughout the study. The same was observed for routine laboratory parameters, including inflammatory markers (C-reactive protein, erythrocyte sedimentation rate) and routine biochemical measurements, including glucose, renal function and lipids, with the only exception of triglycerides which significantly decreased at the end of the study. Before initiation of JAK inhibitors, SEVR was 126 (102–144) %. After three months treatment with JAK inhibitors, calculated SEVR was 134 (106–251) %, which corresponded to a non-significant difference (*p* = 0.083), as presented in Fig. 1.

## Discussion

To the best of our knowledge, this is the first study investigating the effect of treatment with JAK inhibitors on coronary microvascular function in patients with RA. Although preliminary, results of the present study do not support a significant impact of JAK inhibition on myocardial perfusion in treated patients with RA. Importantly, the study population presented with a cluster of cardiovascular risk factors (age ≥ 65 years, history of current or past smoking, other cardiovascular risk factors such as diabetes, obesity, hypertension), which would have rendered them more vulnerable to adverse cardiovascular effects according to the EULAR recommendations and EMA safety warnings. The present study findings further imply that potential cardiovascular aspects of JAK inhibition may be elicited through other mechanisms directly or indirectly affecting the cardiovascular system, rather than directly affecting coronary blood flow [12].

Despite credible safety concerns raised from the ORAL surveillance study [2], the impact of JAK inhibition on cardiovascular health in treated patients with RA remains under investigation. A meta-analysis of pooled data from a total of 215,278 patients revealed no statistically significant difference for major adverse cardiovascular events [15]. Likewise, recent real-world data appear rather reassuring with cardiovascular outcomes largely dependent on the accumulation of cardiovascular risk factors [16, 17, 18]. Concordantly, in a nationwide cohort study of 4,230 RA patients without baseline cardiovascular disease, JAK inhibitors were not associated with the occurrence of major adverse cardiovascular events compared to biological DMARDs [19]. Increasing evidence from real-world prospective studies and narrative literature reviews consistently points to a null impact of JAK inhibitors on the cardiovascular system at least among individuals without exaggerated cardiovascular risk at baseline, as some cardiovascular metabolic factors are increased while others are decreased following treatment with JAK inhibitors [20, 21].

Results from cohort studies highlight the significant role of coronary microvascular dysfunction as a potential contributor to increased cardiovascular risk and mortality in RA, similar to what has been observed in diabetes mellitus. They also provide evidence of a mechanistic connection between inflammation and cardiovascular disease [22]. Increasing evidence indicates that RA patients without clinically evident coronary microvascular dysfunction exhibit more pronounced endothelial dysfunction compared to matched non-RA controls, even in the early stages of the disease [23]. In another RA cohort with a low prevalence of cardiovascular risk factors, nearly half of the participants exhibited coronary microvascular dysfunction at baseline. While reduction of inflammation was not associated with improved myocardial flow reserve, a modest decrease in interleukin-1b, without involvement of other inflammatory pathways, was linked to a reduction in asymptomatic myocardial injury [24]. Sophisticated immunosuppressive treatments, including JAK inhibitors, may help sustain vascular function by reducing inflammation. Findings from basic research suggest that JAK inhibition may help preserve cardiovascular function to some extent by suppressing vascular inflammation [25]. Remarkably, the JAK/STAT pathway has been implicated in the pathogenesis of atherosclerosis, with a causal role of its downstream signaling factors in this process [26].

By contrast, studies assessing *in vivo* vascular effects of JAK inhibitors in patients with RA remain to date extremely limited. As previously published in the same group of patients as of this study, three months’ treatment with JAK inhibitors may induce significant alterations in microvascular capillaroscopic parameters, whereas markers of macrovascular function and morphology, i.e. arterial stiffness and carotid atherosclerosis, remained largely unaffected [12]. Consistent with these results, one-year tofacitinib treatment did not affect arterial stiffness in a larger cohort of 30 RA individuals. Although JAK inhibition effectively suppressed systemic inflammation and improved functional status, robust markers of endothelial function (flow-mediated dilation and asymmetric dimethylarginine) remained unchanged and further increases in carotid intima-media thickness were observed at follow-up [27]. Our study adds to the existing literature by suggesting that myocardial perfusion remains unaltered during treatment with JAK inhibitors in patients with RA. Unless further clinical investigation provides a better understanding of underlying mechanisms, cardiovascular manifestations of JAK inhibitors will remain controversial.

Strengths of the present study include its prospective design and a solid methodological approach using a widely applied, noninvasive index of myocardial perfusion. The study is limited by the absence of a control group and the small sample size, which does not allow for appropriate adjustment for potential confounders, such as disease activity and inflammation, that may interfere with SEVR values as previously shown [10]. In addition, a larger population might have been able to reveal statistically significant differences in SEVR. As follow-up was limited to three months, long-term cardiac effects of JAK inhibitors were not assessed. It is possible that treatment with JAK inhibitors, or any other drug, requires a prolonged period of treatment to induce significant changes in SEVR. In a previous study of 31 patients with RA emerging from a primary cohort who were about to start anti-inflammatory treatment, SEVR did not change over a follow-up period of 12 months [10]. Hence, results of the present study may be considered as preliminary unless verified from larger studies.

In conclusion, three-month treatment with JAK inhibitors did not affect coronary microvascular blood flow in the present cohort of RA patients, as captured non-invasively *in vivo* with SEVR. Future studies are warranted to clarify whether treatment with JAK inhibitors exerts any direct or indirect cardiac effects in patients with RA, specify the duration of treatment potentially associated with such effects, and clearly define the profile of patients who are mostly at risk.
